# The Social and Emotional Well-being of Indigenous Peoples Living With Diabetes: A Systematic Review Protocol

**DOI:** 10.3389/fcdhc.2022.902395

**Published:** 2022-06-30

**Authors:** Jane Speight, Shaira Baptista, Christopher Lee, Louisa Sher, Timothy C. Skinner, Alex Brown

**Affiliations:** ^1^ School of Psychology, Deakin University, Geelong, VIC, Australia; ^2^ The Australian Centre for Behavioural Research in Diabetes, Diabetes Victoria, Melbourne, VIC, Australia; ^3^ First Nations Diabetes consumer and advocate, Melbourne, VIC, Australia; ^4^ Library, Deakin University, Geelong, VIC, Australia; ^5^ LaTrobe University, Melbourne, VIC, Australia; ^6^ College of Health & Medicine, Australian National University, Canberra, ACT, Australia; ^7^ Telethon Kids Institute, Perth, WA, Australia

**Keywords:** type 2 diabetes, indigenous people, social and emotional wellbeing, mental health, systematic review protocol

## Abstract

**Introduction:**

Globally, Indigenous people have a greater incidence and earlier onset of diabetes than the general population and have higher documented rates of emotional distress and mental illness. This systematic review will provide a synthesis and critical appraisal of the evidence focused on the social and emotional well-being of Indigenous peoples living with diabetes, including prevalence, impact, moderators, and the efficacy of interventions.

**Methods:**

We will search MEDLINE Complete, EMBASE, APA PsycINFO, and CINAHL Complete from inception until late April 2021. Search strategies will include keywords related to Indigenous peoples, diabetes, and social and emotional well-being. All abstracts will be rated independently by two researchers against specified inclusion criteria. Eligible studies will report social and emotional well-being data for Indigenous people with diabetes, and/or report on the efficacy of interventions designed to address social and emotional well-being in this population. For each eligible study, quality will be rated using standard checklists to appraise each study’s internal validity, to be determined based on study type. Any discrepancies will be resolved through discussions and consultation with other investigators as needed. We expect to present a narrative synthesis of the evidence.

**Discussion:**

The findings of the systematic review will enable greater understanding of the impact of relationships between diabetes and emotional well-being among Indigenous peoples to inform research, policy and practice. The findings will be accessible to Indigenous people affected by diabetes through a summary published in plain language on our research centre’s website.

**Systematic Review Registration:**

PROSPERO registration number: CRD42021246560.

## Introduction

Indigenous populations, throughout the world, experience lower life expectancy than their non-Indigenous counterparts (1, 2). For example, in Australia, Indigenous people live ten years less than the national average, and those living in remote communities live fifteen years less than the general Australian population (1). The primary contributors to lower life expectancy are the high incidence and early onset of chronic health conditions, such as diabetes (3–5). For example, in the US, diabetes prevalence is almost double among American Indians and Alaskan Natives when compared with Caucasians (6). In Canada, among First Nations people, the lifetime risk of developing diabetes is twice that of their non-Indigenous counterparts, and this higher risk is prevalent at a younger age (7). Indigenous peoples across the globe experience a higher rate of diabetes-related complications, hospitalization and mortality due to diabetes, and concomitant lower life expectancy (6–10).

The profound emotional burden of living with diabetes is well recognized in the contemporary literature on this topic. For example, the ‘45 and Up’ study found that, among both Aboriginal people (N=1,631) and non-Aboriginal people (N=231,774), psychological distress was more likely among those with diabetes than without (11). However, there is limited understanding of the social and emotional burden of living with diabetes among Indigenous peoples. It is recognized widely that mental health and psychological well-being are more than the absence of specific mental disorders, such as depression/anxiety (12). For Indigenous people, the construct of ‘social and emotional well-being’ is paramount, reflecting the more collective and relational world views that are held by Indigenous people with respect to health (13, 14). A social and emotional well-being perspective holds that the individual self is inseparable from the social and ecological system of family, kinship, community, and ancestors, framed within a deeply spiritual connection Indigenous peoples have to land (14–17). Whilst this concept arises from an Aboriginal Australian and Torres Strait Islander world view, there is alignment with the perspectives of many other Indigenous populations, such as the Native American Indians and Alaska Natives, First Nations populations in Canada, Native Hawaiians, Māori, and Sámi communities (13, 16, 18–20). Therefore, cultural ways of knowing, being, and doing, are central to maintaining strong connections to these domains. As a consequence, health and wellbeing more broadly, go beyond the definitions proposed by the WHO and similar organisations (21). Critically important is that social and emotional well-being considers an individual’s historical, political, and social context and that health is shaped by a range of important factors that exist beyond the individual. Thus, when considering social and emotional well-being, we need to consider the continuing effects of colonisation, dispossession, intergenerational historical trauma, forced separation from family and culture, and ongoing systemic racism and social exclusion (14, 22, 23). Therefore, it is essential to include all these dimensions when considering the impact of diabetes on Indigenous populations, not just the commonly reported data on depression and anxiety.

Among non-Indigenous people, reviews show that living with diabetes, with or without associated complications, is associated with significant psychological distress (24). Approximately 42% of Australian adults with diabetes live with medium-to-high rates of psychological distress (25). Further, depressive disorders increase the risk of developing type 2 diabetes and individuals with diabetes have a higher risk of developing depression (26, 27). A study of the prevalence of depression in Aboriginal Australians with type 2 diabetes found that one quarter had current depression compared with 11% of Anglo Celt Australians (28). Although there appears to be a difference in the prevalence of psychological distress between Indigenous and non-Indigenous peoples, the factors associated with such distress may or may not be similar (11). Thus, there remain numerous unanswered questions about the (sub-optimal) social and emotional well-being of Indigenous people with diabetes with regard to etiology, epiphenomena, prevention and treatment.

In addition to general psychological distress, which can be caused by numerous factors, diabetes-specific distress is common among non-Indigenous people with diabetes. Diabetes distress is the emotional distress related to the burden, regimen and interpersonal factors involved in daily management of diabetes (29). Whilst the literature on diabetes-specific distress indicates that there is much commonality in the experience of diabetes distress around the world, the research has not comprehensively incorporated the experiences of Indigenous populations. Given the continuing colonization, land dispossession, individual and systemic discrimination and racism inflicted on Indigenous populations, and the impacts of multiple intergenerational traumas, it is not possible to extrapolate the data from the dominant westernized distress perspectives to Indigenous peoples with diabetes.

Our aim is to conduct a systematic review of the social and emotional well-being of Indigenous peoples with diabetes, and develop recommendations for future research, clinical practice and health policy. Our specific objectives are to consolidate and critically appraise the evidence examining among Indigenous peoples with diabetes:

- the experience of social and emotional well-being, including the prevalence of psychological distress (including depressive symptoms, anxiety symptoms, diabetes-specific distress);
- variations in this experience by characteristics, such as type of diabetes and treatment regimen, presence of diabetes complications, gender, age;
- the impact, e.g. on diabetes self-care (e.g. physical activity, diet, medication-taking), and physical health indicators (e.g. glycated hemoglobin (HbA1c));
- potential moderators of generic and diabetes-specific distress
- the availability and effectiveness of interventions designed to mitigate or reduce psychological distress or enhance social and emotional well-being.

## Materials and Methods

This protocol was prepared using the Preferred Reporting Items for Systematic review and Meta-Analysis Protocols (PRISMA-P) guidelines (30). Conduct of the systematic review began in April 2021 and will likely be completed by September 2022.

### Eligibility Criteria

Studies will be considered for inclusion in the review according to a PICOS strategy:

#### Population

Indigenous people of any age, living with diabetes (all types) in the continents of Australia, Europe and North America. These continents were selected given that their history of European settlement/colonization, whereby Indigenous people are no longer the dominant cultural group.

#### Intervention or Exposure

For trials, pre-post and some cross-sectional studies, the intervention is expected to be designed to reduce or mitigate psychological distress. For experiments and trials, the exposure is expected to be the experience of psychological distress.

#### Comparison or Control Groups

For trials, pre-post and some cross-sectional studies, we will include any comparator, including usual care, waitlist control or active treatment conditions; however, studies will be eligible even where there is no comparison/control.

#### Outcomes

Social and emotional well-being (operationalized as positive well-being but also negative constructs, such as depression, anxiety). Diabetes-specific outcomes will also be included, e.g. diabetes distress. Where distress is the predictor or mediating variable, then the outcome of interest may be any related to the experience of living with diabetes (e.g. self-efficacy, self-care behaviors, medication taking, physical health indicators).

#### Study Design and Setting

Articles with quantitative data will be eligible for inclusion if they report empirical research, including but not limited to randomized and non-randomized trials, pre-post studies, observational and cohort studies, cross-sectional surveys. Qualitative studies will be excluded from the current review but considered for a separate analysis if sufficient studies are identified. Studies conducted in Australia, New Zealand, Europe, North America or Hawaii will be included.

We will exclude non-peer reviewed articles, abstracts without full-text available, studies published in languages other than English, reviews, commentaries, and editorials and qualitative studies. In addition, studies not reporting the data needed to calculate effect sizes will be excluded from any meta-analyses conducted if the data cannot be sourced directly from authors.

### Search Strategy

A systematic search will be conducted in: MEDLINE Complete (via EBSCOhost), EMBASE (Via EMBASE), APA PsycINFO (via EBSCOhost), and CINAHL Complete (via EBSCOhost). Searches will focus on the main concepts of diabetes, Indigenous people and well-being. For a draft of the MEDLINE Complete search strategy see **Supplementary File 1**. The search will be designed by a Health Liaison Librarian with experience in systematic search methods in collaboration with the subject experts on the team. The search will be proofed by another librarian using the PRESS checklist (31). The search will not be limited to any publication date, language or publication type, however papers not written in English or not adequately translated by Google translate may be excluded due to resource limits for translations. Citation searching for citations and references of key articles will be performed in Scopus. Grey Literature will not be included in the review.

Two researchers will further develop and pilot the search strategy, with critical input from other investigators, in consultation with our Deakin Librarians who will check our search strategy (above) using the Evidence-Based Checklist for the Peer Review of Electronic Search Strategies (PRESS EBC) (31). Following confirmation of the search strategy, but before the search is conducted, the review protocol will be registered in PROSPERO (CRD42021246560).

### Study Selection

Using the Cochrane Collaboration’s COVIDENCE software, two researchers will screen the titles and abstracts independently and eliminate those not meeting the eligibility criteria. Full-text articles will be obtained of positively screened titles/abstracts as well as those where eligibility is uncertain from the information provided in the abstract. Two researchers will screen all full-text articles independently and eliminate those not meeting the eligibility criteria. At both stages, discrepancies will be resolved through mutual agreement and consultation with other investigators as needed.

### Data Extraction

Data will be extracted from eligible studies using a pre-specified template, to be developed by the researchers specifically for this review. Information to be extracted is likely to include but will not be limited to: authors and publication year, country, study design, sample size, participant characteristics (e.g. age, gender, diabetes type, diabetes duration and treatment regimen), measures, and results (including associations with other variables of interest).

### Quality Assessment/Risk of Bias

The quality of each eligible study will be rated using standard checklists to appraise each study’s internal validity, to be determined based on study type in accordance with published guidelines, including NHMRC guidelines (32), the CONSIDER statement (33) and the CREATE tool (34).

Any discrepancies will be resolved through discussions and consultation with other investigators as needed.

### Evidence Synthesis

For the reporting of results, we will follow the PRISMA-P guidance (30) and include a flowchart of the process, and summary tables. We will describe the nature of the studies identified, in terms of characteristics such as study design, country of origin, sample size, and the diabetes type and gender of participants. Where possible, we will report on the prevalence of psychological distress, the experience, associations with other outcomes of interest, mediators of psychological distress and moderators of impact, and effective interventions to reduce or mitigate psychological distress.

If possible, meta-analysis will be conducted to examine prevalence of psychological distress (e.g., across countries, by gender), and impact of psychological distress on physical health outcomes (e.g., HbA1c). Meta-analysis will include forest plots and examination of between study statistical heterogeneity using the I^2^ statistic.

Most likely, we will conduct a narrative synthesis describing the characteristics of the studies and the main findings of interest. We will regard the p<0.05 as statistically significant, unless the authors of the included studies have applied more conservative values. Where possible, we will report on the prevalence of psychological distress in the population. All reported relationships between psychological distress and other participant characteristics or other outcomes of interest will be included in the synthesis, regardless of whether these are reported in tables or text (and regardless of whether they are positive/negative, statistically significant or not). In addition, we will consider covariates, where reported, in these relationships. If possible, meta-analysis will be conducted to examine prevalence of psychological distress (e.g., across countries, by gender), and impact of psychological distress on physical health outcomes (e.g. HbA1c). We recognize that meta-analysis may not be possible, given the potential heterogeneity of outcome measures used to assess social and emotional well-being in this population. Depending on the number of included studies identified and their scope, we may synthesize data separately according to each of our stated objectives or include them all in one synthesis. If sufficient qualitative studies are identified, a separate synthesis will be conducted following established methods outlined in Thomas and Harden (35): “Methods for thematic synthesis of qualitative research in systematic reviews” (35).

If the data allow, subgroup analyses may be conducted by country of origin, type of diabetes, or type of outcome (e.g., depressive symptoms vs diabetes distress).

### Ethics and Dissemination

This study does not require ethics approval as it does not involve new empirical research with human or animal participants.

### Patient and Public Involvement

Indigenous people with diabetes are included in our investigator team, were involved in developing our research questions and are authors of this manuscript. AB identifies as a member of the Yuin Nation.

## Discussion

Understanding the social and emotional well-being of Indigenous peoples living with diabetes is crucial to improve the policy, resources, and support options available to this population who experience considerable disadvantage. Hence, this protocol introduces a systematic review to consolidate and critically appraise the current evidence. To our knowledge, this is the first protocol to describe a systematic review to examine the social and emotional well-being of Indigenous peoples living with diabetes across the world. Key strengths of this review are that it will be conducted according to PRISMA guidance, and that it is led by a team of researchers experienced in both the emotional well-being of Indigenous people and of non-Indigenous people with diabetes. In addition, the review will ensure robust assessment of methodological quality of the included studies to ascertain the validity of their findings and to ensure that risk of bias is minimised. The restriction of studies to those published in the English language may be a limitation.

The evidence generated from this systematic review will add a new perspective that will be useful to inform future research regarding the social and emotional well-being of Indigenous peoples living with diabetes.

## Author Contributions

JS, CL, TS and AB conceived the study and research questions. They prepared the review protocol with input from LS who prepared the search strategy. JS and SB prepared the first draft of the manuscript, which was reviewed by all authors for critical content. All authors approve the final version.

## Funding

This research is supported by an unrestricted grant from Diabetes Australia. The funding body is not involved in the conduct or reporting of the research. JS is supported by core funding to the Australian Centre for Behavioural Research in Diabetes provided by the collaboration between Diabetes Victoria and Deakin University.

## Conflict of Interest

The authors declare that the research was conducted in the absence of any commercial or financial relationships that could be construed as a potential conflict of interest.

## Publisher’s Note

All claims expressed in this article are solely those of the authors and do not necessarily represent those of their affiliated organizations, or those of the publisher, the editors and the reviewers. Any product that may be evaluated in this article, or claim that may be made by its manufacturer, is not guaranteed or endorsed by the publisher.
