# Supplementary material for: The Social and Emotional Well-being of Indigenous Peoples Living With Diabetes: A Systematic Review Protocol
Source: Front Clin Diabetes Healthc. 2022 Jun 30;3:902395. doi: 10.3389/fcdhc.2022.902395 (PMC10012064; doi:10.3389/fcdhc.2022.902395)
Supplement: Supplementary file 1 [file Table_1.docx]

Supplementary Material

# Supplementary File 1

MEDLINE Complete preliminary search design

| **Line** | **Search** |
| --- | --- |
| S15 | S13 AND S8 AND S14  **[All wellbeing AND diabetes AND Indigenous]** |
| S14 | S9 OR S10 OR S11 OR S12  **[Indigenous - AU NZ Nth.Am. EU]** |
| S13 | S1 OR S2 OR S3 OR S4 OR S5 OR S6 OR S7  **[All wellbeing]** |
| S12 | (TI (Indigenous  OR Native or “First Nation*” or “First People*” or Tribe* or Tribal or Nation or Nations OR Shaman OR Saami or Sampi or Sami or Samis or Southernsami* or Umesami* or Pitesami* or Lulesami* or Northernsami* or Enaresami* or Kolasami* or Lapp or Lapps or Lappish or Lappland or Lapland* or Lappalainen* or Saamelainen* or  Lappbys or Samebys or reinbeitesdistrikt or paliskunta or siida) OR AB (Indigenous  OR Native or “First Nation*” or “First People*” or Tribe* or Tribal or Nation or Nations OR Shaman OR Saami or Sampi or Sami or Samis or Southernsami* or Umesami* or Pitesami* or Lulesami* or Northernsami* or Enaresami* or Kolasami* or Lapp or Lapps or Lappish or Lappland or Lapland* or Lappalainen* or Saamelainen* or  Lappbys or Samebys or reinbeitesdistrikt or paliskunta or siida) OR MH "Sami (people)")  AND  (TI (Fennoscandia or Finnmark or Scandinavia or Nordic or Sweden or Norway or Finland or Swedish or Finnish or Norwegian or Norge or Svensk* or Suomi or “Barents Region” or Kola or “Arctic Europe*” or “Polar Europe*” or “North* Europ*”) OR AB (Fennoscandia or Finnmark or Scandinavia or Nordic or Sweden or Norway or Finland or Swedish or Finnish or Norwegian or Norge or Svensk* or Suomi or “Barents Region” or Kola or “Arctic Europe*” or “Polar Europe*” or “North* Europ*”) OR MH Finland OR MH Norway OR MH Sweden)  **[Northern European Indigenous concept]** |
| S11 | (TI (“North America*” OR “United States” OR USA OR American OR Hawaii* OR Canada* OR Canadian) OR AB (“North America*” OR “United States” OR USA OR American OR Hawaii* OR Canada* OR Canadian) OR MH “United states+” OR MH “Canada+”)  AND  (TI (Native or Indigenous or “First Nation*” or “First People*” or Tribe* or Tribal or Indian* or Nation or Nations or Islander* or Inuit* or Aborigin*) OR AB (Native or Indigenous or “First Nation*” or “First People*” or Tribe* or Tribal or Indian* or Nation or Nations or Islander* or Inuit* or Aborigin*) OR MH “Indians, North American” OR MH “Alaska Natives” OR MH “Inuits” OR MH “Oceanic Ancestry Group”)  **[North American Indigenous concept - incl. Canada, Alaska, Hawaii]** |
| S10 | (TI (“New Zealand*” or Northland or Auckland or Waikato or “Bay of Plenty” or Gisborne or “Hawke’s Bay” or Taranaki or Whanganui or Manawatu or Wellington or Marlborough or Nelson or Tasman or “West Coast” or Canterbury or Otago or Southland) OR AB (“New Zealand*” or Northland or Auckland or Waikato or “Bay of Plenty” or Gisborne or “Hawke’s Bay” or Taranaki or Whanganui or Manawatu or Wellington or Marlborough or Nelson or Tasman or “West Coast” or Canterbury or Otago or Southland) OR MH “New Zealand”)  AND  (TI (Aborigin* or “First People*” or “First Nation*” or Indigenous or Native* or Maori or Iwi or Hapu or Tribe* or Tribal or Islander* or Nation or Nations) OR AB (Aborigin* or “First People*” or “First Nation*” or Indigenous or Native* or Maori or Iwi or Hapu or Tribe* or Tribal or Islander* or Nation or Nations)OR MH “Oceanic Ancestry Group”)  **[New Zealand Indigenous Concept]** |
| S9 | (TI (Australia* or Tasmania* or Victoria* or “New South Wales” or Queensland* or “Northern Territory” or “Western Australia*” or “South Australia*” or “Australian Capital Territory”) OR AB (Australia* or Tasmania* or Victoria* or “New South Wales” or Queensland* or “Northern Territory” or “Western Australia*” or “South Australia*” or “Australian Capital Territory”) OR MH Australia+)  AND  (TI (Aborigin* or “First People*” or “First Nation*” or Indigenous or Native* or Islander* or "Torres Strait Islander*" or Tribe* or Tribal or Nation or Nations OR ATSI) OR AB (Aborigin* or “First People*” or “First Nation*” or Indigenous or Native* or Islander* or "Torres Strait Islander*" or Tribe* or Tribal or Nation or Nations OR ATSI) OR MH “Oceanic Ancestry Group”)  **[Australian Indigenous concept]** |
| S8 | TI (NIDDM OR NIDD OR “non insulin* depend*” OR T2DM OR T1DM OR Diabet* ) OR AB (NIDDM OR NIDD OR “non insulin* depend*” OR T2DM OR T1DM OR Diabet* ) OR MH "Diabetes Mellitus+"  **[Diabetes]** |
| S7 | TI (decoloniz* OR decolonis* OR coloniz* OR colonis* OR "cultural safety" OR "social context*" OR "cultur* context*" OR "cultur* identit*" OR "cultur* competen*" OR "cultur* sensitiv*" OR "harmony with nature" OR "cultural practice" OR "cultural continu*" OR "systemic marginal*" OR "community ownership" OR racis* OR discriminat*)  OR AB (decoloniz* OR decolonis* OR coloniz* OR colonis* OR "cultural safety" OR "social context*" OR "cultur* context*" OR "cultur* identit*" OR "cultur* competen*" OR "cultur* sensitiv*" OR "harmony with nature" OR "cultural practice" OR "cultural continu*" OR "systemic marginal*" OR "community ownership" OR racis* OR discriminat*)  OR MH “Social Marginalization” OR MH “Social discrimination” OR MH dehumanization OR MH prejudice OR MH Racism OR MH “health status disparities” OR MH “healthcare disparities”  **[Indigenous cultural terms from gold set]** |
| S6 | TI (optimist* OR optimism OR "positive affect*" OR Mindfulness) OR AB (optimist* OR optimism OR "positive affect*" OR Mindfulness) OR MH mindfulness  **[Positive psychology terms from gold set]** |
| S5 | TI ((substance OR drug* OR alcohol*) N2 (use* OR misuse OR "mis-use" OR abus* OR addiction* or consum*) OR addiction*) OR AB ((substance OR drug* OR alcohol*) N2 (use* OR misuse OR "mis-use" OR abus* OR addiction* or consum*) OR addiction*) OR MH “substance-related disorders” OR MH smoking+ OR MH “substance abuse, intravenous” OR MH ethanol OR MH “opioid-related disorders+” OR MH “behavior, addictive” OR MH “illicit drugs+” OR MH “Cocaine-Related Disorders” OR MH “analgesics, opioid” OR MH “alcohol-related disorders” OR MH “Phencyclidine Abuse” OR MH “Marijuana Abuse” OR MH “inhalant abuse” OR MH “Amphetamine-Related Disorders” OR MH methadone OR MH narcotics OR MH “alcoholic intoxication” OR MH “Binge Drinking” OR MH Alcoholism OR MH “tobacco use disorder” OR MH “risk taking+”  **[Drug use terms from gold set ]** |
| S4 | TI ("psychological factor*" OR "social determinant*" OR "socio-economic" OR socioeconomic OR "social issue*") OR AB ("psychological factor*" OR "social determinant*" OR "socio-economic" OR socioeconomic OR "social issue*") OR MH “mental health” OR MH “social class” OR MH “socioeconomic factors” OR MH “social determinants of health” OR MH poverty  **[Wellbeing terms from gold set]** |
| S3 | TI (angry OR anger OR grief OR griev* OR bereav* OR mood* OR happy OR happiness) OR AB (angry OR anger OR grief OR griev* OR bereav* OR mood* OR happy OR happiness) OR MH emotions+  **[Emotional terms from Gold set]** |
| S2 | TI (worry* OR worrie* OR "social* isolat*" OR (life N2 satisfaction) OR QOL OR "self injur*" OR “good life”) OR AB (worry* OR worrie* OR "social* isolat*" OR (life N2 satisfaction) OR QOL OR "self injur*" OR “good life”)  **[Further Wellbeing terms identified in scoping]** |
| S1 | TI (Wellbeing OR "well being" OR “diabetes distress” OR "social support*" OR "social condition*" OR "social barrier*" OR "social exclu*" OR "social factor*" OR "social stress*" OR ((social OR socio*) N5 health) OR resilien* OR emotion* OR stress* OR distress* OR cope* OR coping OR trauma* OR psychosocial OR “psycho-social” OR psychosocio* OR "community involve*" OR "self determination" OR depressi* OR anxi* OR (quality N2 (life OR living)) OR vulnerab* OR "self care" OR "socio cultural" OR sociocultural OR spiritual* OR wellness OR suicid* OR trust* OR compassion* OR (support N3 (social OR socio* OR psychosocial OR emotion* OR community OR cultur*))) OR AB (Wellbeing OR "well being" OR “diabetes distress” OR "social support*" OR "social condition*" OR "social barrier*" OR "social exclu*" OR "social factor*" OR "social stress*" OR ((social OR socio*) N5 health) OR resilien* OR emotion* OR stress* OR distress* OR cope* OR coping OR trauma* OR psychosocial OR “psycho-social” OR psychosocio* OR "community involve*" OR "self determination" OR depressi* OR anxi* OR (quality N2 (life OR living)) OR vulnerab* OR "self care" OR "socio cultural" OR sociocultural OR spiritual* OR wellness OR suicid* OR trust* OR compassion* OR (support N3 (social OR socio* OR psychosocial OR emotion* OR community OR cultur*))) OR MH “quality of life” OR MH “Psychosocial Functioning” OR MH depression OR MH “stress, psychological” OR MH “mood disorders” OR MH “depressive disorder” OR MH “depressive disorder, major” OR MH ”Dysthymic Disorder” OR MH “anxiety disorders” MH “stress disorders, post-traumatic” OR MH “adaptation, psychological” OR MH “emotional adjustment” OR MH survivorship OR MH “self-injurious behaviour+” OR MH “social support+” OR MH “personal satisfaction” OR MH “social isolation” OR MH “interpersonal relations” OR MH “self concept” OR MH “self disclosure” OR MH “social interaction” OR MH “social integration”  **[Socio Emotional Wellbeing terms from gold set]** |
